# Hospitalisations related to administration errors of psychotropic drugs: a nationwide retrospective study between 1998 and 2019 in Australia

**DOI:** 10.3389/fphar.2023.1149500

**Published:** 2023-06-22

**Authors:** Fatemah M. Alsaleh, Abdallah Y. Naser, Zahra K. Alsairafi, Richard Ofori-Asenso

**Affiliations:** ^1^ Department of Pharmacy Practice, College of Pharmacy, Kuwait University, Hawalli, Kuwait; ^2^ Department of Applied Pharmaceutical Sciences and Clinical Pharmacy, Faculty of Pharmacy, Isra University, Amman, Jordan; ^3^ School of Public Health and Preventive Medicine, Monash University, Melbourne, VIC, Australia

**Keywords:** admission, Australia, hospitalisation, medication, psychotropic, poisoning, administration error

## Abstract

**Objectives:** Medication administration error occurs when there is a discrepancy between what the patient received or was planned to receive and what the doctor originally intended. The aim of this study was to examine the trends in hospitalisation related to administration errors of psychotropic drugs in Australia.

**Materials and Methods:** This was a secular trend analysis study that examined the hospitalisation pattern for medication administration errors of psychotropic drugs in Australia between 1998 and 2019. Data on medication administration errors of psychotropic drugs was obtained from The National Hospital Morbidity Database. We analysed the variation in hospitalisation rates using the Pearson chi-square test for independence.

**Results:** Hospitalisation rates related to administration errors of psychotropic drugs increased by 8.3% [from 36.22 (95% CI 35.36—37.08) in 1998 to 39.21 (95% CI 38.44—39.98) in 2019 per 100,000 persons, *p* < 0.05]. Overnight-stay hospital admission patients accounted for 70.3% of the total number of episodes. Rates of same-day hospitalisation increased by 12.3% [from 10.35 (95% CI 9.90—10.81) in 1998 to 11.63 (95% CI 11.21—12.05) in 2019 per 100,000 persons]. Rates of overnight-stay hospital admission increased by 1.8% [from 25.86 (95% CI 25.13—26.59) in 1998 to 26.34 (95% CI 25.71—26.97) in 2019 per 100,000 persons]. Other and unspecified antidepressants (selective serotonin and norepinephrine reuptake inhibitors) were the most common reason for hospitalisation accounting for 36.6% of the total number of hospitalisation episodes. Females accounted for 111,029 hospitalisation episodes, representing 63.2% of all hospitalisation episodes. The age group 20–39 years accounted for nearly half (48.6%) of the total number of episodes.

**Conclusion:** Psychotropic drug administration error is a regular cause of hospitalization in Australia. Hospitalizations usually required overnight stays. The majority of hospitalizations were in persons aged 20–39 years, which is concerning and warrants further investigation. Future studies should examine the risk factors for hospitalization related to psychiatric drug administration errors.

## Introduction

Any error made when prescribing, dispensing, or administering a medication is referred to as a medication error. The definition of a medication administration error (MAE) is any deviation between what the patient received or was supposed to receive and what the doctor intended when the order was placed (Zed et al., 2008). MAE arises when there is a difference between the medicine obtained by the patient and the desired drug therapy (Williams et al., 2007). According to the ICD system MAEs include poisoning by, adverse effect of, and underdosing of medications (International Classification of Diseases, 2023). Drug poisoning is potentially one of the major drug-related problems and the primary reason for patient admission to critical care units and emergency departments worldwide (Kurdil, 2021). It is the main cause of non-traumatic coma among patients under the age of 35 admitted to the emergency department (Demİrel, 2010). In developed countries, the yearly poisoning incidence rate ranges from 0.02% to 0.93%, and this rate is increasing globally (Özayar et al., 2011).

Worldwide, acute poisoning is a prominent cause of mortality and morbidity and a frequent reason for hospital admissions and emergency service visits (Jesslin et al., 2010; Rutto et al., 2012; Maheswari et al., 2016). Besides, the most frequent global reason for acute poisoning is drug overdose. Poisoning by psychotropic drugs, including benzodiazepines, antidepressants, and antipsychotics is the most common method by which individuals attempt suicide (Moens et al., 1989; Kim et al., 2015; Mowry et al., 2015). The toxicity of psychotropic medicines varies greatly, ranging from acute to chronic (Bickel, 1986). Although the real impact of psychotropic medications differs across countries, it is typically a severe health issue requiring an urgent attention (Kinoshita et al., 2015).

Women are approximately twice as likely to suffer from mood and anxiety disorders, while men are approximately four times more likely to suffer from substance-use and impulsive disorders (Offord et al., 1996; Seedat et al., 2009). The gender-based difference in the prevalence of psychiatric disorders itself contributes to the difference in the utilization of psychotropic medications and ultimately their associated MAEs. A previous study in the United Kingdom (United Kingdom) reported that the hospitalization rate for psychotropic drug poisoning increased in England and Wales by 20.0% between the periods from 1999 to 2020 (Al-Daghastani and Naser, 2022). In Australia, intentional self-poisoning with psychotropic, anti-parkinsonism, sedative-hypnotic, and anti-epileptic drugs was the most frequent form of self-harm leading to hospital admissions between 2008/2009 and 2020/2021, accounting for 40% of intentional self-harm hospital admissions in 2020/2021 (Australian Institute of Health and Welfare, 2022a). Given the risks associated with psychotropic drug use and poisoning, it is crucial to identify the patterns of hospitals admissions due to administration errors relevant to these agents. This will help understanding the extent of the problem and to advise strategies for reducing and preventing patient harm. Therefore, this study aimed to examine the trends of hospitalisation related to administration errors of psychotropic drugs in Australia.

## Material and methods

### Study design

This was a secular trend analysis study that examined the hospitalisation pattern for medication administration errors of psychotropic drugs in Australia between 1998 and 2019.

### Data sources

#### National hospital morbidity database

The National Hospital Data Collection (NHDC) covers the National Hospital Morbidity Database (NHMD). The Australian Institute of Health and Welfare (AIHW) maintains several key national hospital databases, which are included in the NHDC (Australian Institute of Health and Welfare, 2022b). The NHMD, an online database, receives data from Australia’s state and territory health authorities (Australian Institute of Health and Welfare, 2022c). The data gathered at the NHMD is made up of sets of episode-level information from morbidity data collection systems of patients admitted to private and public hospitals in Australia. The data are based on the NMDS for admitted patient care and contain information on the patients’ diagnoses, external sources of injury and poisoning, length of hospital stays, treatments received, and demographics. The goal of NMDS for admitted patient care is to collect data on the treatment offered to hospitalized patients in Australian hospitals. The NMDS includes episodes of care for individuals admitted to hospitals from all alcohol and drug treatment facilities, independent day hospitals, and private and public mental and acute hospitals. Using the ICD-10, we identified hospitalization events related to all medication administration errors of psychotropic drugs (T43). The data included in this study are for patients who were primarily admitted to hospitals due to medication administration errors of psychotropic drugs (poisoning by, adverse effect of and underdosing of medications). Same-day hospitalisation is defined as “a day during which a person admitted as an inpatient is confined to a bed and in which the patient stays overnight in a hospital”. Overnight-stay admitted care is defined as “the care provided for a minimum of one night, to a patient who is admitted to and separated from the hospital on different dates”. Differentiating whether a patient will require an overnight stay or will be admitted on the same day is essential for the development of appropriate treatment plans. Longer hospital visits may necessitate additional assessments, interventions, and monitoring, whereas same-day admissions may be subject to specific care protocols or post-procedure instructions.

### Australian Bureau of Statistics

Mid-year population data between 1998 and 2019 was collected from The Australian Bureau of Statistics (ABS) (Australian Bureau of Statistics, 2022a). Between 1998 and 2016, data on the population were collected using the historical population (Australian Institute of Health and Welfare, 2022d). Between 2017 and 2019, population data were collected utilizing national, state, and territorial populations (Australian Bureau of Statistics, 2016).

### Study population

From 1998 to 2019, data on all private and public hospitalizations in Australia were collected for this study (Australian Bureau of Statistics, 2022b).

### Statistical analysis

SPSS version 27 (IBM Corp, Armonk, NY, United States) was used for all analyses. Hospitalisation rates with 95% CIs were determined by dividing hospitalisation episodes by the mid-year population. Pearson chi-square test for independence was used to analyse the variation in hospitalisation rates between 1998 and 2019. The confidence interval was estimated using the following equation for the population proportion: *p*^ +/− z*(*p*^(1 − *p*^)/n)^0.5.

## Results

### Administration errors of psychotropic drugs hospitalisation

Between 1998 and 2019, there were 176,925 episodes of hospitalisation for administration errors of psychotropic drugs reported in Australia. The total yearly number of hospitalisation episodes increased by 45.8% from 6,813 in 1998 to 9,935 in 2019, representing an increase in hospitalisation rate of 8.3% [from 36.22 (95% CI 35.36—37.08) in 1998 to 39.21 (95% CI 38.44—39.98) in 2019 per 100,000 persons, *p* < 0.05].

**FIGURE 1:**
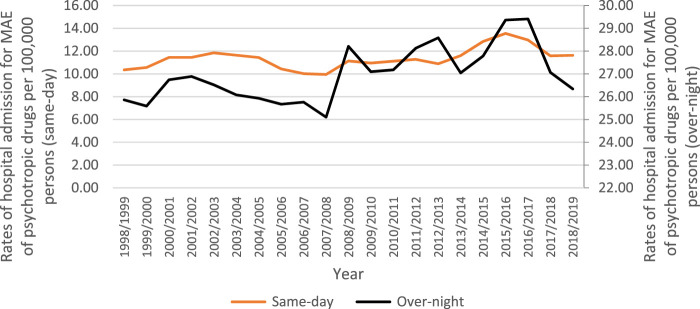
Rates of same-day and overnight-stay patients hospital admission in Australia between 1998 and 2019.

A total of 70.3% of all hospitalization episodes involved patients admitted for an overnight stay, while 29.7% involved same-day admissions. Rates of same-day hospitalisation increased by 12.3% [from 10.35 (95%CI 9.90—10.81) in 1998 to 11.63 (95%CI 11.21—12.05) in 2019 per 100,000 persons]. Rates of overnight-stay hospital admission increased by 1.8% [from 25.86 (95%CI 25.13—26.59) in 1998 to 26.34 (95%CI 25.71—26.97) in 2019 per 100,000 persons] (Figure 1).

Other and unspecified antidepressants (selective serotonin and norepinephrine reuptake inhibitors) were the most common reason for administration errors of psychotropic drugs hospitalisation accounting for 36.6% of the total number, followed by other and unspecified antipsychotics and neuroleptics with 29.3%, psychostimulants with potential for use disorder with 11.6%, tricyclic and tetracyclic antidepressants with 11.2%, and phenothiazine antipsychotics and neuroleptics with 9.4% (Table 1).

**TABLE 1:** Percentage of hospitalisation from total number of hospitalisation episodes per ICD code.

ICD code	Description	Percentage from total number of hospitalisation episodes (%)
T43.0	“Tricyclic and tetracyclic antidepressants”	11.2
T43.1	“Monoamine-oxidase-inhibitor antidepressants”	0.7
T43.2	“Other and unspecified antidepressants (selective serotonin and norepinephrine reuptake inhibitors)”	36.6
T43.3	“Phenothiazine antipsychotics and neuroleptics”	9.4
T43.4	“Butyrophenone and thioxanthene neuroleptics”	0.7
T43.5	“Other and unspecified antipsychotics and neuroleptics”	29.3
T43.6	“Psychostimulants with potential for use disorder”	11.6
T43.8	“Other psychotropic drugs, not elsewhere classified”	0.5
T43.9	“Psychotropic drug, unspecified”	0.1

### Trends of hospitalisation (based on indication)

During the study period, administration errors of psychotropic drugs hospitalisation rate for other and unspecified antipsychotics and neuroleptics rose considerably by 361.6%. Moreover, hospitalisation rate for psychostimulants with potential for use disorder increased by 73.3%. However, hospitalisation rate for other psychotropic drugs, unspecified, butyrophenone and thioxanthene neuroleptics, monoamine-oxidase-inhibitor antidepressants, phenothiazine antipsychotics and neuroleptics, other and unspecified antidepressants, and tricyclic and tetracyclic antidepressants decreased by 100.0%, 100.0%, 79.6%, 72.4%, 65.6%, 23.4%, and 8.8%, respectively (Table 2; Supplementary Figure S1).

**TABLE 2:** Percentage change in the hospitalisation rates from 1998—2019 in Australia.

Poisonings	Hospitalisation rate in 1998 per 100,000 persons (95% CI)	Hospitalisation rate in 2019 per 100,000 persons (95% CI)	Percentage change from 1998—2019 (%)
“Tricyclic and tetracyclic antidepressants”	3.69 (3.41—3.96)	3.37 (3.14—3.59)	−8.8
“Monoamine-oxidase-inhibitor antidepressants”	0.41 (0.32—0.51)	0.11 (0.07—0.16)	−72.4
“Other and unspecified antidepressants (selective serotonin and norepinephrine reuptake inhibitors)”	16.61 (16.03—17.19)	12.73 (12.29—13.17)	−23.4
“Phenothiazine antipsychotics and neuroleptics”	7.53 (7.13—7.92)	2.59 (2.39—2.79)	−65.6
“Butyrophenone and thioxanthene neuroleptics”	0.64 (0.52—0.75)	0.13 (0.09—0.17)	−79.6
“Other and unspecified antipsychotics and neuroleptics”	3.20 (2.94—3.46)	14.77 (14.30—15.24)	361.6
“Psychostimulants with potential for use disorder”	3.17 (2.92—3.43)	5.50 (5.21—5.79)	73.3
“Other psychotropic drugs, not elsewhere classified”	0.88 (0.75—1.02)	0.00 (0.00—0.00)	−100.0
“Psychotropic drug, unspecified”	0.08 (0.04—0.12)	0.00 (0.00—0.00)	−100.0

### Trends in hospitalisation stratified by gender

Females accounted for 111,029 hospitalisation episodes, representing 63.2% of all hospitalisation episodes, with a mean number of 5,287 episodes per year. Hospitalisation rate among females increased by 10.3% [from 44.02 (95% CI 42.69—45.36) in 1998 to 48.55 (95% CI 47.34—49.76) in 2019 per 100,000 persons]. Hospitalisation rate among males decreased by 3.8% [from 28.30 (95% CI 27.22—29.38) in 1998 to 27.21 (95% CI 26.30—28.12) in 2019 per 100,000 persons] (Supplementary Figure S2).

### Trends in hospitalisation stratified by age group

As per age group differences in hospitalisation episodes, the persons aged 20–39 years accounted for 48.6% of the total number of episodes, followed by the group aged 40–59 years with 26.9%, the age group below 20 years with 19.5%, the age group 60–74 years with 3.7%, and then the age group 75 years and above with 1.3%. The highest increase in the hospitalisation rate was observed among patients aged below 20 years. On the other hand, the hospitalisation rate among patients aged 20–39 years decreased by 10.5%, Table 3. The trends of hospitalisation rate stratified by age group is presented in Supplementary Figure S3.

**TABLE 3:** Percentage change in hospitalisation rate stratified by age.

Age group	Hospitalisation rate in 1998 per 100,000 persons (95% CI)	Hospitalisation rate in 2019 per 100,000 persons (95% CI)	Percentage change (%)
Below 20 years	20.35 (95%CI 19.13—21.58)	32.74 (95%CI 31.32—34.16)	60.9
20–39 years	66.41 (95%CI 64.28—68.53)	59.42 (95%CI 57.65—61.19)	−10.5
40–59 years	35.47 (95%CI 33.80—37.15)	40.03 (95%CI 38.48—41.59)	12.9
60–74 years	9.30 (95%CI 7.98—10.62)	14.19 (95%CI 12.98—15.41)	52.6
75 years and above	8.32 (95%CI 6.55—10.09)	9.31 (95%CI 7.88—10.74)	11.9

### Trends in hospitalisation stratified by indication and gender

The preponderance of MAE of psychotropic drugs, not elsewhere classified hospital admission rates were higher among females compared to males, that include the following: tricyclic and tetracyclic antidepressants, monoamine-oxidase-inhibitor antidepressants, other and unspecified antidepressants, phenothiazine antipsychotics and neuroleptics, butyrophenone and thioxanthene neuroleptics, and other and unspecified antipsychotics and neuroleptics (Supplementary Figure S1). Still, MAE of psychotropic drugs, not elsewhere classified hospital admission rates for psychostimulants with potential for use disorder, other psychotropic drugs, not elsewhere classified, and psychotropic drug, unspecified were higher among males compared to females (Supplementary Figure S4).

### Trends in hospitalisation stratified by indication and age

All MAE of psychotropic drugs, not elsewhere classified-related hospital admission rates were more common among the age group 20–39 years (Supplementary Figure S5).

## Discussion

To our knowledge, this is the first study to utilise a nationwide database in Australia to describe the patterns of hospital admission due to psychotropic drug administration errors. Results from the current study showed that hospitalisation rate for administration errors of psychotropic drugs has increased in Australia by 8.3% during the periods from 1998 to 2019, with an annual growth of 45.8%. One explanation for this growth could be the rise in mental illnesses and hence psychotropic prescribing practices in Australia (Wallis et al., 2021; Pai et al., 2022) and worldwide (Rani et al., 2008; Lao et al., 2017). Regardless, this increase imposes an urgent attention to be explored and monitored to prevent future patient harm and reduce costs (Suh et al., 2000; Leendertse et al., 2011). It is estimated that approximately 44% of Australians aged 16–85 have experienced a mental disorder at some point in their lives, with 21% having experienced a mental disorder in the preceding 12 months 17% of Australians suffer from anxiety disorders, followed by 8% with affective disorders and 3% with substance use disorders (Australian Institute of Health and Welfare, 2023). In addition, a recent study in Australia by Bartholomaeus et al. found that between 2009 and 2019, psychological therapy claims and GP mental health treatments increased by 167.4% and 85.4%, respectively (Bartholomaeus et al., 2023).

In line with results from the current study, a study in the United Kingdom showed that the hospital admission rate due to MAE of psychotropic medications increased by 19.9% over 21 years’ study period (Al-Daghastani and Naser, 2022). Similar growth in the recorded number of administration errors of psychotropic drugs was reported in the United States by Vargas et al. (2020). On the other hand, a higher increase was noted in the United States of America in 2010 by Coben et al. (2010), where approximately a two-third (65%) increase in hospitalisation rate was reported. This higher increase can be related to the broader scheme of drugs used in Coben’s study which also included opioids and sedatives. In the current study, the overnight-stay administration errors of psychotropic drugs hospital admission patients accounted for most hospitalisation episodes (70.3%) as compared to one-third of the episodes that were for same day patients. This indicates the seriousness of adverse effects caused by these agents (Bickel, 1986; Kinoshita et al., 2015; Chan, 2019).

The most common cause of administration errors of psychotropic drugs that lead to hospital admission in the current study was MAE of other and unspecified antidepressants, which accounted for 36.6% of the total number of hospitalisation episodes. This was followed by other and unspecified antipsychotics and neuroleptics, psychostimulants with potential for use disorder, tricyclic and tetracyclic antidepressants, and phenothiazine antipsychotics and neuroleptics which accounted for 29.3%, 11.6%, 11.2% and 9.4%, respectively. These finding were consistent to the study in the United Kingdom where the main cause of increased hospital admission was mainly caused by unspecified poisoning with antidepressants which accounted for 48.9%, followed by MAE of tricyclic and tetracyclic antidepressants, and unspecified poisoning with antipsychotics and neuroleptics which accounted for 20.9% and 13.4%, respectively (Al-Daghastani and Naser, 2022). Although overdosing on tricyclic and tetracyclic antidepressants can be hazardous, the percentage of hospital admissions due to these agents were less in the current study in comparison to the study in the United Kingdom. This can be related to the fact that these agents are not recommended as first-line treatment. In addition, prescribing practices in Australia showed that only 25% of patients are prescribed these agents as compared to 52% of antidepressant prescriptions which where the less toxic agents in overdose SSRIs were chosen as first-line agents (Malhi et al., 2022).

With regards to gender, females were responsible for the majority of hospitalisation episodes (63.2%) in the current study. Females’ hospital admission rate has increased by 10.3% while hospital admission rates among males have decreased by 3.8% over the years between 1998 and 2019. In this context, studies in the literature have clearly documented that females are more likely than males to be poisoned by psychotropic drugs (Vargas et al., 2020). This could be explained by the higher prevalence of psychiatric disorders (e.g., depression and anxiety) among female patients as compared to the males and hence receiving a higher number of psychotropic prescriptions (Boyd et al., 2015). In an Australian study by Malhi et al. (2022), antidepressants prescribing patterns were investigated between the years of 3,013 and 2019 and it was found that more than half of the study sample (56%) were female patients.

The age range 20–39 years in the current study accounted for almost half (48.6%) of the total number of hospitalization episodes, followed by the age group 40–59 years with 26.9%. On the other hand, children and younger adults (below age of 20 years) showed a lower admission rate of 19.5%. This could be explained in part by the higher usage of psychotropic medications among older age groups (Malhi et al., 2022), adding to the fact that children and adolescents more likely to be poisoned by over-the-counter medications as compared to prescription medications (Huynh et al., 2018).

In the current study, the overnight-stay administration errors of psychotropic drugs hospital admission patients accounted for most hospitalisation episodes (70.3%) as compared to one-third of the episodes that were for same day patients. However, future research would be beneficial to investigate the risk factors for longer hospital stay. With regards to age groups, future studies might include investigating the prevalence trends of different classes of psychotropics per each age group. Exploring rate of admission due to psychotropic poisoning based on diagnosed conditions is another point to be considered by future studies.

To the best of our knowledge, this is the first study to examine the trends of hospitalisation related to administration errors of psychotropic drugs in Australia. Our study was not restricted to a specific age group or gender which increase the generalisability of our findings. At the same time, this study has limitations. The data used in this study was on the population level not on the individual level of the patients which restricted our ability to identify risk factors that might have influenced our estimated hospitalisation rate. Our hospitalisation rate estimates include re-admission episodes, which might have led to an overestimation. Therefore, our findings should be interpreted carefully.

In conclusion, psychotropic drugs administration errors are common reason for hospitalization in Australia. Most hospitalisation episodes required an overnight stay. The majority of hospitalizations were in persons aged 20–39 years, which is an alarming result that requires additional investigation. Future studies should examine the risk factors for hospitalization related to psychiatric medication poisoning.

## Data Availability

Publicly available datasets were analyzed in this study. This data can be found here: https://meteor.aihw.gov.au/content/394352.
