# Supplementary material for: Hospitalisations related to administration errors of psychotropic drugs: a nationwide retrospective study between 1998 and 2019 in Australia
Source: Front Pharmacol. 2023 Jun 22;14:1149500. doi: 10.3389/fphar.2023.1149500 (PMC10323131; doi:10.3389/fphar.2023.1149500)
Supplement: Supplementary file 1 [file DataSheet1.docx]

Supplementary material:

Figure S1: Rates of hospital admission in Australia stratified by type between 1998 and 2019.

Figure S2: Hospitalisation rates in Australia stratified by gender between 1998 and 2019.

Figure S3: Hospital admission rates in Australia stratified by age group between 1998 and 2019.


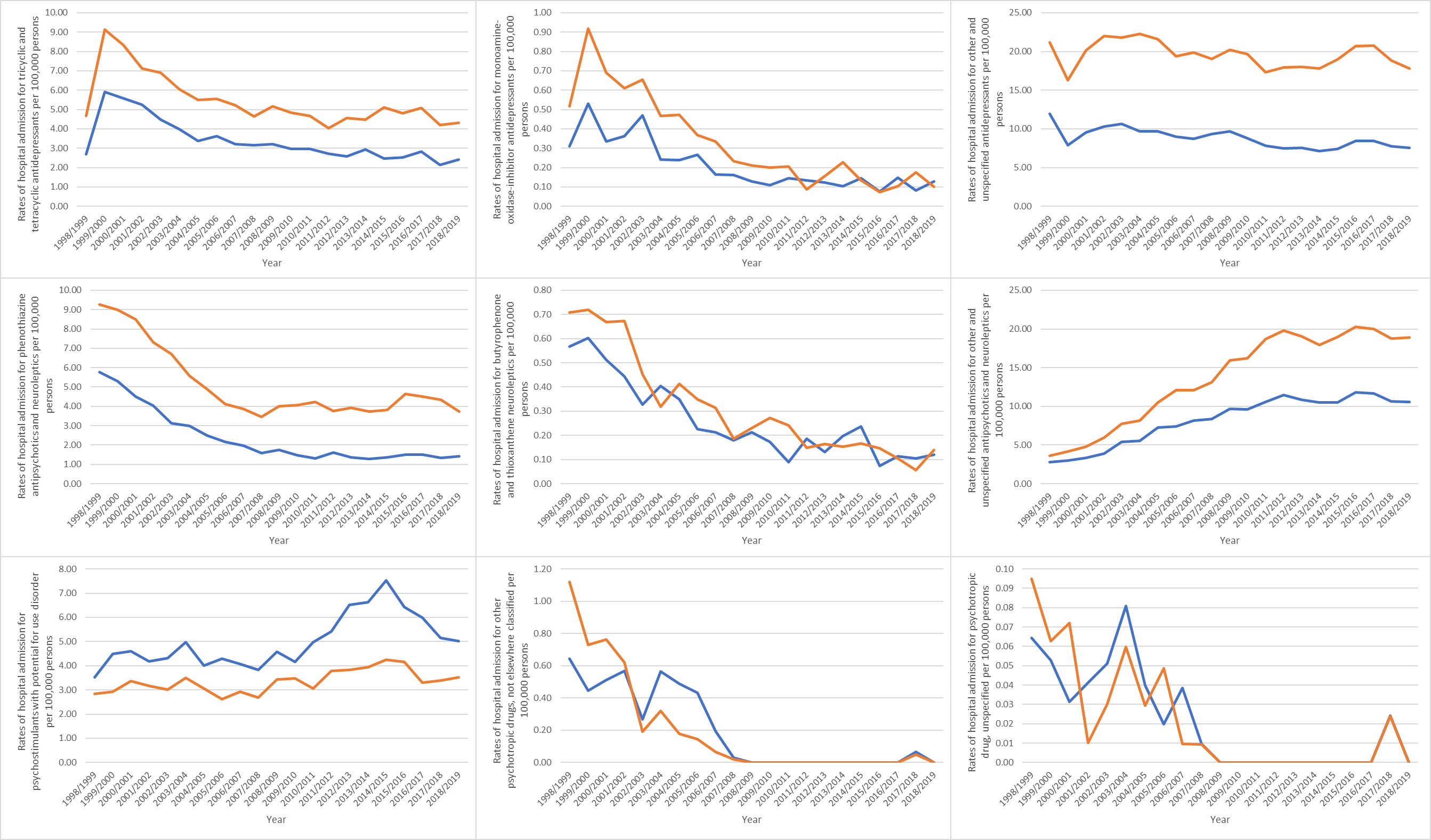


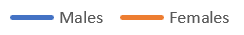
Figure S4: Rates of hospitalisation in Australia stratified by indication and gender between 1998 and 2019

**
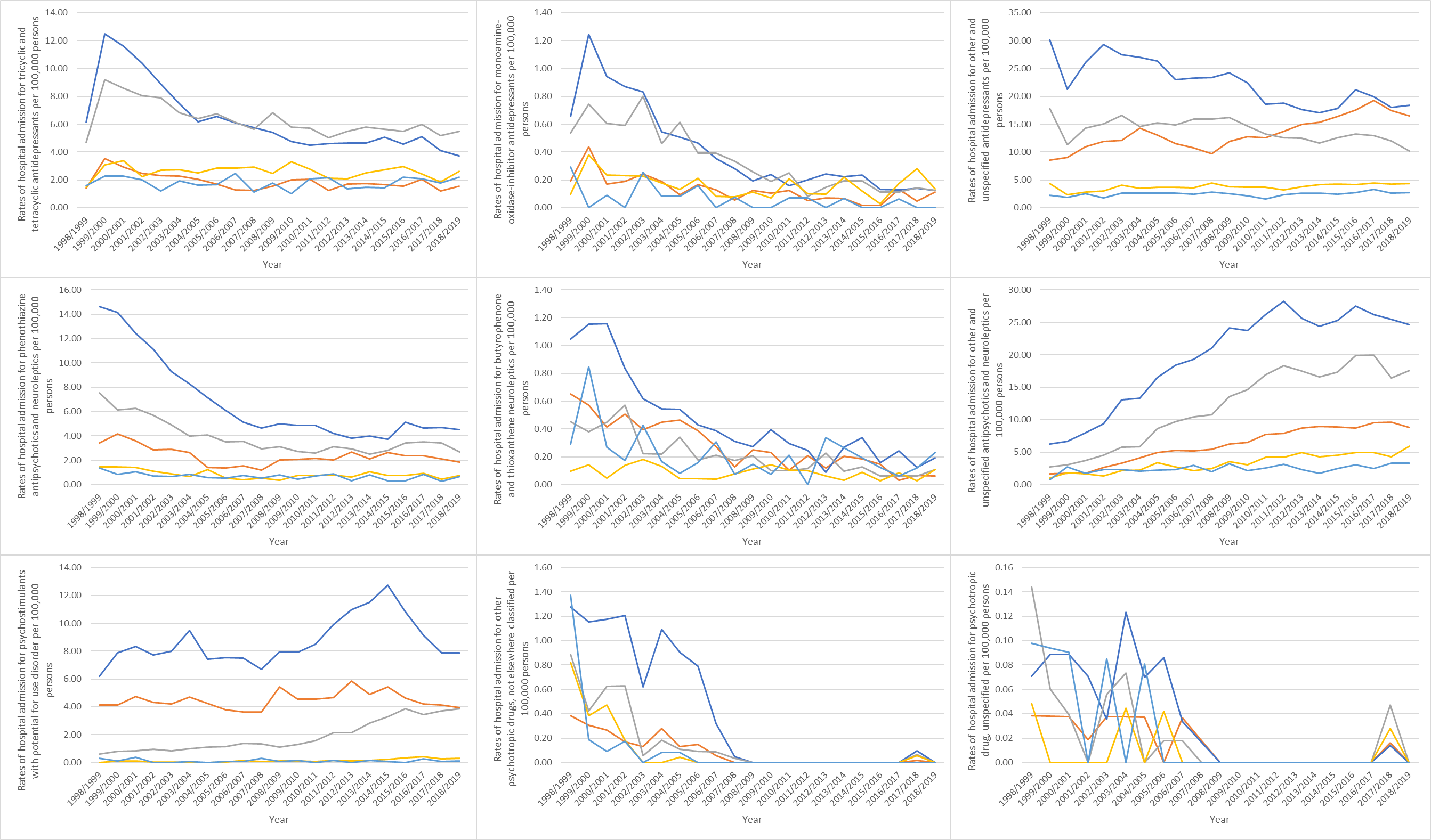
**


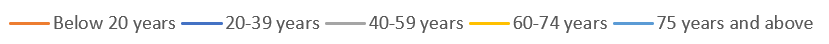
Figure S5: Rates of hospitalisation in Australia stratified by indication and age group between 1998 and 2019.
